# The best of hepato-pancreato-biliary (HPB) surgery in *BJS Open*: advancing frontiers towards 2025

**DOI:** 10.1093/bjsopen/zrae167

**Published:** 2025-02-20

**Authors:** Giovanni Marchegiani

**Affiliations:** Editor

Among the science published in *BJS Open*, the year 2024 witnessed remarkable contributions on hepato-pancreato-biliary (HPB) surgery that have the potential to shape some specific aspects of the current surgical practice and pave the way for future research endeavours.

Starting from the liver, the article ‘Importance of resection margin after resection of colorectal liver metastases in the era of modern chemotherapy: population-based cohort study’^1^ by Östrand *et al*. addresses an old but gold technical and oncological issue. The authors demonstrate that, despite the advent of modern chemotherapeutic regimens, achieving a resection margin of ≥1 mm remains a significant prognostic factor for overall survival in patients undergoing resection for colorectal liver metastases. As for other areas of surgical oncology, this finding underscores the importance of meticulous surgical technique and highlights the need for continued refinement of operative strategies to ensure optimal oncological outcomes. It is somehow ‘relieving’ for surgeons that such a simple but important issue is reappraised. Our job is, and remains, primarily practical and skill dependent. This article elegantly reinforces that no compromises can be made with a strict surgical technique, ever.

Remaining in the field of surgical technique but moving to the pancreas, ‘Impact of the radiological morphology of the mesopancreas on the outcome after pancreatoduodenectomy for pancreatic ductal adenocarcinoma: retrospective study’^2^ by Navez *et al*. delves into the intricate relationship between the radiological appearance of the mesopancreas and surgical outcomes in patients undergoing pancreatoduodenectomy for pancreatic ductal adenocarcinoma. The authors demonstrate that solid infiltration of the mesopancreas, as observed on preoperative imaging, is associated with poorer disease-free and overall survival. The main take home of this article is two-fold. Again, the technical implications of a correct mesopancreas dissection, the core of a pancreatoduodenectomy, are reinforced. Moreover, the prognostic value of thorough radiological assessments should guide treatment strategies, including the consideration of neoadjuvant therapies. In the era of multimodal treatments and multidisciplinary treatment strategy, this key factor must be emphasized.

We finish where we started, back on the liver with ‘Outcome of the novel description of arterial position changes after major liver resections: retrospective study’^3^ by Abbasi Dezfouli *et al*. The article introduces a novel perspective on postoperative complications following major liver resections. The authors describe and classify arterial position changes, specifically involving the coeliac trunk and superior mesenteric artery, and their association with increased morbidity and mortality rates. This thought-provoking work opens new avenues for understanding the underlying mechanisms of postoperative complications and may inform preventive and therapeutic strategies, such as prophylactic arterial stenting in high-risk patients. Is this truly the way forward? More evidence is needed, but once again it is reassuring how the application of the meticulous study of anatomy can be applied to improving surgical outcome.

This selection of articles nicely exemplifies the ongoing pursuit of excellence in HPB surgery, addressing diverse aspects of patient care, from surgical technique and perioperative risk assessment to radiological evaluation and postoperative management. Their collective impact lies in the potential to refine clinical decision-making, optimize surgical outcomes, and stimulate further research to address unmet needs in this complex and continuing evolving field. The editors have in particular appreciated that, as we look ahead, these contributions serve as stepping stones for future investigations. Prospective studies are warranted to validate the findings, develop standardized protocols and explore novel therapeutic interventions. Multidisciplinary collaborations among surgeons, radiologists, pathologists and oncologists will be instrumental in translating these insights into tangible improvements in patient care.
